# Biomedical concept recognition with error-aware negative-enhanced ranking framework

**DOI:** 10.1093/bioinformatics/btag495

**Published:** 2026-07-03

**Authors:** Shanshan Liu, Noriki Nishida, Fei Cheng, Takehito Utsuro, Yuji Matsumoto

**Affiliations:** RIKEN Center for Advanced Intelligence Project, Nihonbashi 1-chome Mitsui Building, 15th floor, 1-4-1 Nihonbashi, Chuo-ku, Tokyo, 103-0027, Japan; Graduate School of Science and Technology, University of Tsukuba, 1-1-1 Tennodai, Tsukuba, Ibaraki, 305-8577, Japan; RIKEN Center for Advanced Intelligence Project, Nihonbashi 1-chome Mitsui Building, 15th floor, 1-4-1 Nihonbashi, Chuo-ku, Tokyo, 103-0027, Japan; Graduate School of Informatics, Kyoto University, S212, Research Bldg. No.9, Yoshida Main Campus, Yoshida-honmachi, Sakyo-ku, Kyoto, 606-8501, Japan; Faculty of Engineering, Information and Systems, University of Tsukuba, 1-1-1 Tennodai, Tsukuba, 305-8573, Japan; RIKEN Center for Advanced Intelligence Project, Nihonbashi 1-chome Mitsui Building, 15th floor, 1-4-1 Nihonbashi, Chuo-ku, Tokyo, 103-0027, Japan

## Abstract

**Motivation:**

Mention-agnostic biomedical concept recognition (MA-BCR) requires inferring ontology concepts directly from passages, without relying on explicit mention spans. Prior work has mainly focused on generative and classification-based approaches. Ranking-based methods typically use a retrieve-rerank pipeline, and this paradigm has not been systematically studied for MA-BCR. Consequently, it remains unclear how ranking-based approaches compare with existing paradigms and what types of supervision are most beneficial for ranker training under limited annotation settings.

**Results:**

Through a systematic comparison of ranking-, generative-, and classification-based paradigms, we show that a two-stage retrieve-rerank architecture is the most robust and scalable backbone for MA-BCR. Building on this finding, we propose **ENR**, an error-aware negative-enhanced ranking framework that augments training with false positives collected from heterogeneous recognizers, improving reranking performance without increasing inference-time cost. Experiments on MM-HPO and MM-GO (two datasets derived from MedMentions-ST21pv) demonstrate that ENR substantially outperforms prior approaches.

**Availability and implementation:**

The code and data underlying this article are available in Github at https://github.com/sl-633/enr-recognizer or in Zenodo at https://doi.org/10.5281/zenodo.20730803.

## 1 Introduction

Biomedical concept recognition (BCR) refers to the automatic identification of ontology concepts in unstructured text. It has been studied in several biomedical domains, including phenotype concept recognition (Luo *et al.* 2021, Groza *et al.* 2024), clinical concept recognition in electronic health records (Fu *et al.* 2020, Lossio-Ventura *et al.* 2022), and large-scale indexing of biomedical articles with controlled vocabularies such as MeSH (Jin *et al.* 2018, Xun *et al.* 2019, Rae *et al.* 2020). These studies demonstrate that automatically mapping free text to ontology concepts is both feasible and practically important, supporting downstream tasks such as patient phenotype profiling, clinical information extraction, and semantic retrieval of the literature (Funk *et al.* 2014, Jin *et al.* 2018, Luo *et al.* 2021).

Traditional BCR systems assume that each relevant concept is realized through explicit textual mentions. Such systems typically identify mention spans using named entity recognition (NER) and then link each span to an ontology entry using entity linking models (Kolitsas *et al.* 2018, De Cao *et al.* 2020, Wu *et al.* 2020). These approaches are effective when concepts have clear surface forms, but they fail when concepts are only implicitly described within scientific or clinical narratives (El Khettari *et al.* 2024). For instance, many biological processes in the Homeostasis Imbalance Process (HOIP) ontology do not appear verbatim in passages (Yamagata *et al.* 2024). When no specific mention exists, mention-dependent pipelines cannot recover the correct ontology concepts, leaving a substantial gap in current biomedical text mining workflows.

This limitation motivates mention-agnostic biomedical concept recognition (MA-BCR), in which systems identify ontology concepts directly from passages without a mention identification step. Target concepts may be explicitly expressed in the passage or implicitly conveyed through distributed contextual evidence. MA-BCR reduces dependence on costly span-level annotation and more closely reflects how human experts infer concepts from implicit or distributed textual cues.

Early MA-BCR studies have explored generative and classification-based approaches, including LLM prompting, supervised generative recognition, and extreme multi-label classification (Zhang *et al.* 2021, El Khettari *et al.* 2024, Liu *et al.* 2025). Despite their success, these paradigms exhibit structural limitations. Generative models, whether prompted or trained, are primarily optimized with positive supervision and are not explicitly penalized for assigning high probability to incorrect concepts, which limits their discriminative ability in large ontologies. Extreme multi-label classifiers can scale to tens of thousands of concepts, but they rely on positive-only label supervision and are sensitive to mismatch between training and inference distributions (Zhang *et al.* 2021, Kharbanda *et al.* 2022). Therefore, existing MA-BCR formulations often struggle either to distinguish the correct concept from many plausible but incorrect ontology candidates or to make effective use of limited supervision when scaled to large biomedical ontologies.

**Ranking-based modelling** represents a promising yet under-explored direction for MA-BCR. This paradigm is widely used in related tasks such as entity linking, information retrieval, and knowledge graph alignment, where models must select the correct item from a large candidate pool (Kolitsas *et al.* 2018, Wang *et al.* 2020, Wu *et al.* 2020). By treating MA-BCR as passage–concept relevance estimation, ranking models in the retrieve-rerank architecture can learn directly from contrasts between relevant and irrelevant concepts while supporting scalable search over large ontologies.

However, ranking-based paradigms have not been systematically evaluated for MA-BCR. Existing studies have explored ranking only in limited forms, such as untrained *k*-nearest-neighbour retrieval over embeddings (Liu *et al.* 2025) or reranking concept names generated by LLM-based recognizers (Shlyk *et al.* 2024), rather than training ranking models to directly estimate passage–concept relevance at ontology scale. Prior work has also included cross-encoder relevance models as supervized baselines (El Khettari *et al.* 2024). Although these models are often described as “classifiers”, they are architecturally ranking-based because they score passage–concept compatibility. However, they are typically trained with pointwise binary objectives and evaluated over restricted candidate sets, rather than with contrastive learning and retrieval-style inference.

Consequently, two questions remain open. First, it remains unclear how ranking-based approaches compare with generative and classification paradigms under realistic ontology-scale MA-BCR settings. Second, although hard negative mining is a central component of modern retrieve-rerank systems, it remains unclear what types of negatives are most beneficial for MA-BCR and whether model-specific error patterns from heterogeneous recognizers can be exploited to improve ranker training.

**Figure 1:**
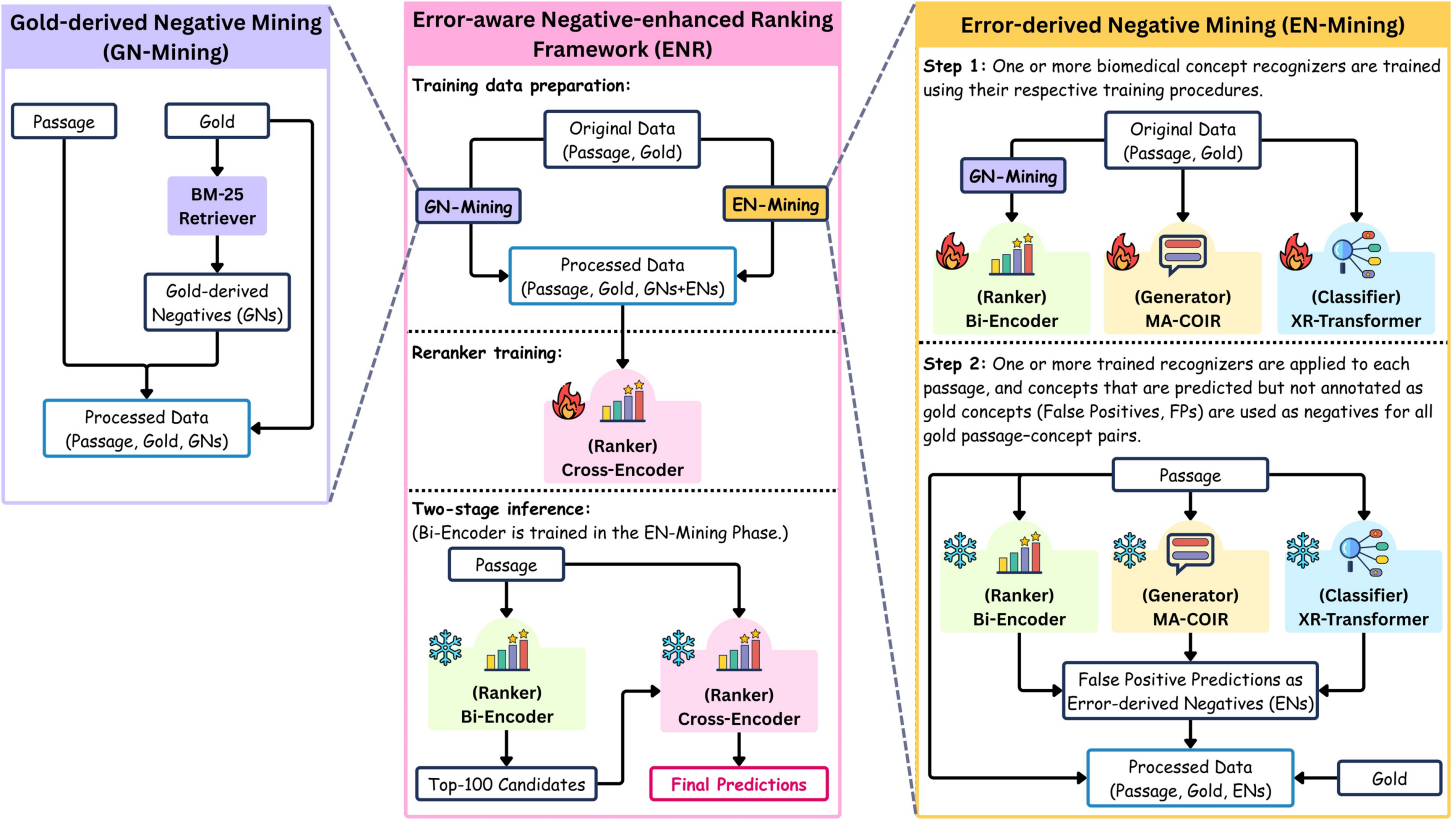
Overview of the error-aware negative-enhanced ranking framework (ENR) for mention-agnostic biomedical concept recognition (MA-BCR). ENR combines gold-derived negatives (GN-mining) and error-derived negatives (EN-mining) to construct enhanced training data for a listwise cross-encoder ranker. EN-mining collects false positive predictions from heterogeneous one-stage biomedical concept recognizers. Inference follows a standard bi-encoder→cross-encoder retrieve-rerank pipeline without additional overhead.

This study addresses these questions through two main contributions. First, we provide the first systematic comparison of generative, classification-based, and ranking-based paradigms for MA-BCR, covering both one-stage and two-stage inference settings. This analysis identifies how the paradigms differ in recall–precision trade-offs and error characteristics, and shows that retrieve-rerank architectures offer the most robust and scalable foundation for MA-BCR. Second, building on these findings, we introduce **ENR**, an **e**rror-aware **n**egative-enhanced **r**anking framework. As illustrated in Fig. 1, ENR leverages false positive predictions from multiple pretrained recognizers, together with lexically similar distractors retrieved by BM25, to enrich negative pools and strengthen contrastive training of cross-encoder rankers. Experimental results show that incorporating error-derived negatives from diverse recognizers provides strong learning signals and leads to substantial performance gains on human phenotype abnormality and biological process concept recognition. Overall, our work both clarifies the modelling landscape of MA-BCR and presents an effective training framework that turns cross-paradigm error signals into improved ranking performance.

## 2 Materials and methods

### 2.1 Task definition

In this work, ontology concepts are treated as independent prediction targets during training and evaluation. We intentionally adopt this formulation to study supervision design for large-label concept recognition without introducing ontology-specific structural assumptions, allowing the framework to be applied consistently to both hierarchical ontologies and flat concept inventories.

Let C denote the target ontology concept set. In mention-agnostic biomedical concept recognition (MA-BCR), the input is a passage p and the goal is to predict the set of relevant concepts $\hat{Y}(p)\subseteq C$ conveyed by p. Equivalently, MA-BCR can be viewed as estimating passage-concept relevance over C via a scoring function s(p,c). Predictions are then obtained by selecting a subset of concepts according to s (e.g. by thresholding or top-*L* selection).

### 2.2 Paradigms and models

**Figure 2:**
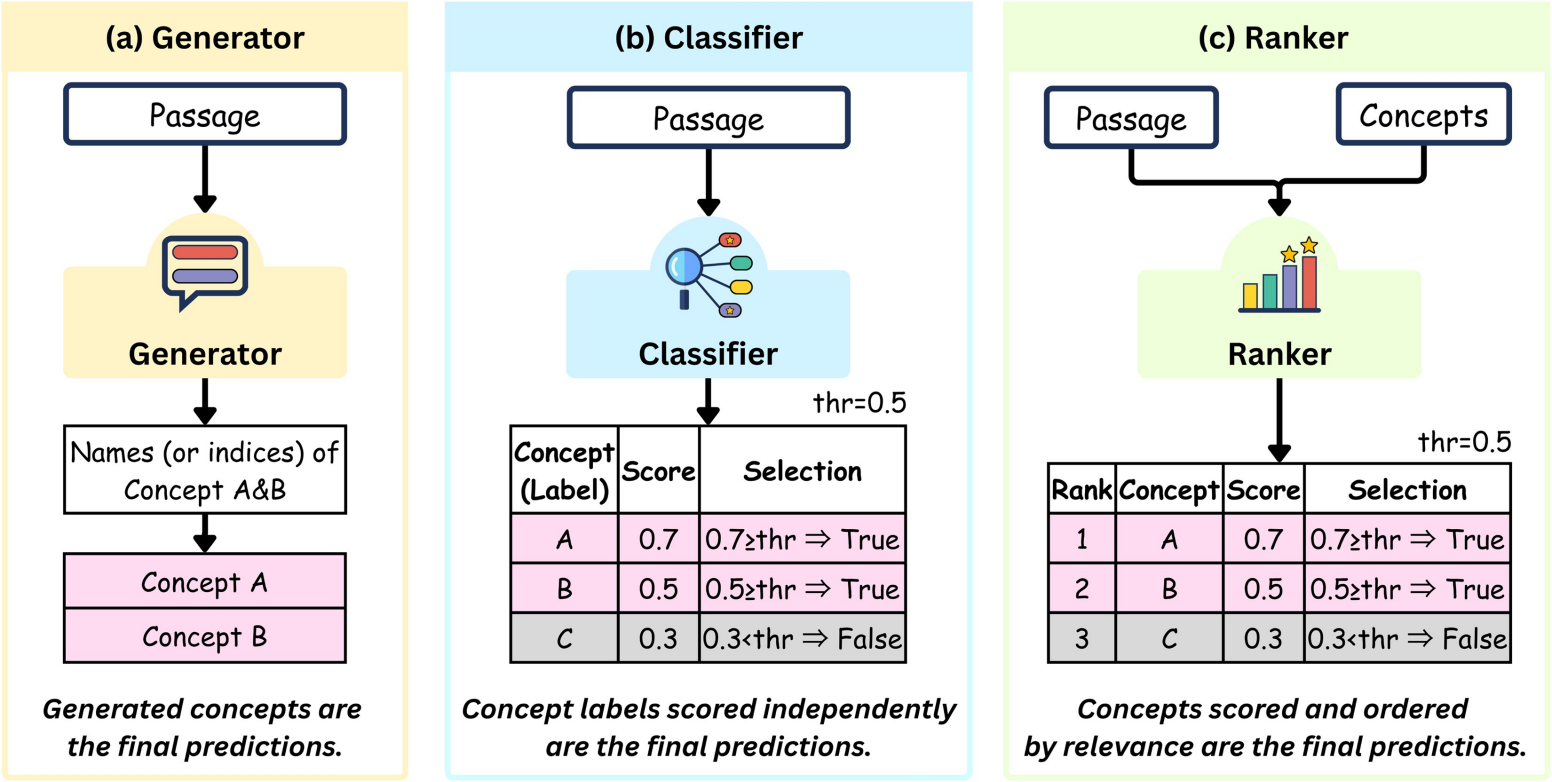
Three one-stage paradigms for MA-BCR. (a) A generator produces concepts (or indices) directly from the passage. (b) A classifier predicts relevant concepts via multi-label classification over the concept label space. (c) A ranker scores and orders concepts with respect to the passage; predictions can be adjusted through a threshold (thr).

To systematically compare modelling paradigms for MA-BCR (Fig. 2), we evaluate three representative paradigms: (a) **Generative-based: MA-COIR** (Liu *et al.* 2025) as a generative one-stage recognizer; (b) **Classification-based: XR-Transformer** (Zhang *et al.* 2021) as an extreme multi-label classifier; and (c) **Ranking-based models**, including a **bi-encoder retriever** (Wu *et al.* 2020) and a **cross-encoder ranker** (Nogueira and Cho 2019). For ranking, we consider both a Pointwise Cross-Encoder Ranker (PCER) baseline and our main Listwise Cross-Encoder Ranker (LCER). These paradigms differ fundamentally in supervision: generators and classifiers are primarily trained with positive supervision, whereas rankers explicitly learn to separate gold concepts from hard negatives via contrastive objectives.

#### 2.2.1 Generator: MA-COIR

We adopt MA-COIR (Liu *et al.* 2025) as a generative one-stage recognizer under an indexing-recognition formulation. Each ontology concept is assigned a structured index, and given an input passage p, a sequence-to-sequence model generates a sequence of one or more concept indices. Decoded indices are then mapped back to ontology concepts via an index–concept lookup table.

Following Liu *et al.* (2026), we use the Semantic Search Index (SSI) to assign a unique index sequence to each concept based on a hierarchical label tree.

*Training and decoding.* MA-COIR is trained by minimizing sequence-level cross-entropy over target SSI sequences. At inference time, decoding is constrained to valid SSI tokens and prefixes permitted by the label tree, ensuring that all generated sequences correspond to valid concepts.

#### 2.2.2 Classifier: XR-Transformer

We utilize XR-Transformer (Zhang *et al.* 2021), which frames MA-BCR as extreme multi-label text classification. Each ontology concept is treated as a label, and a passage p may be assigned multiple labels. XR-Transformer uses a hierarchical label tree to scale prediction to large label spaces by progressively narrowing down candidate labels.

*Training objective.* XR-Transformer optimizes a multi-label classification objective over the label space, learning from positive concept assignments without explicitly constructing passage-concept pairs or contrastive negatives. At inference time, it outputs concept-level scores that can be converted to a prediction set via thresholding or top-*L* selection.

#### 2.2.3 Ranking-based proposal: bi-encoder retriever

The bi-encoder uses a Siamese architecture with a shared-weight Transformer encoder to represent passages and concepts separately, enabling efficient retrieval at scale.

*Encoding.* Given a passage p and a concept c, we construct input sequences as:


(1) $$T_{p}=[\text{CLS}]\;p\;[\text{SEP}],\;T_{c}=[\text{CLS}]\;{\text{Name}}_{c}\;[\text{SEP}],$$

where [CLS] and [SEP] are special tokens. Both sequences are encoded by the same encoder Enc(·) into vectors:


(2) $$H_{p}=\text{Enc}(T_{p})\in R^{|T_{p}|\times d},\;H_{c}=\text{Enc}(T_{c})\in R^{|T_{c}|\times d},$$

where d is the representation dimension. We obtain fixed-size embeddings via mask-aware mean pooling:


(3) $$z_{p}=\text{MeanPool}(H_{p}),\;z_{c}=\text{MeanPool}(H_{c}),$$

and score relevance by cosine similarity,


(4) $$s_{\text{BE}}(p,c)=\text{cos}(z_{p},z_{c}).$$

At inference time, concept embeddings are pre-encoded and indexed, and retrieval is performed via nearest-neighbour search.

*Mini-batch construction.* We apply contrastive learning for ranker-based model training. We construct mini-batches from a pool of *positive passage–concept pairs* $\left(p,c^{+}\right)$; a passage may be paired with multiple positive concepts and therefore can appear in multiple positive pairs. Each mini-batch starts by sampling B positive pairs ${\left\{(p_{i},c_{i}^{+})\right\}}_{i=1}^{B}$, where B is the mini-batch size. For each sampled pair, we sample K negative concepts $\left\{c_{i,1}^{-},\ldots ,c_{i,K}^{-}\right\}$ (with K=2 in our experiments) and aggregate all concepts in the batch into a candidate set


$$C_{\text{cand}}=\overset{B}{\underset{i=1}{\cup }}\left\{c_{i}^{+},c_{i,1}^{-},\ldots ,c_{i,K}^{-}\right\}.$$

*Training objective.* For each $p_{i}$, we apply a softmax cross-entropy loss over $C_{\text{cand}}$, treating $c_{i}^{+}$ as the correct class:


(5) $$L_{\text{BE}}=-\sum_{i=1}^{B}\;\text{log}\;\frac{\;\text{exp}\;\left(s_{\text{BE}}(p_{i},c_{i}^{+})\right)}{\sum_{c\in C_{\text{cand}}}\;\text{exp}\;\left(s_{\text{BE}}(p_{i},c)\right)}.$$

This objective uses both sampled negatives and in-batch negatives (i.e. concepts contributed by other sampled pairs in the same mini-batch).

#### 2.2.4 Ranking-based proposal: cross-encoder ranker

The cross-encoder jointly encodes a passage and a concept with a single Transformer encoder, allowing cross-attention between the passage and the concept name.

*Encoding.* Given a passage p and a concept c, we form the paired input:


(6) $$T_{p,c}=[\text{CLS}]\;{\text{Name}}_{c}\;[\text{SEP}]\;p\;[\text{SEP}],$$

where [CLS] and [SEP] are special tokens.

The paired input $T_{p,c}$ is fed into a Transformer encoder Enc(·) to obtain representations:


(7) $$H_{p,c}=\text{Enc}(T_{p,c})\in R^{n\times d},$$

where n is the sequence length of $T_{p,c}$.

Let $h_{\left[\text{CLS}\right]}\in R^{d}$ denote the row of $H_{p,c}$ corresponding to the [CLS] token. We map it to a scalar score with a linear head:


(8) $$s_{\text{CE}}(p,c)=w^{⊤}h_{\left[\text{CLS}\right]}+b.$$

At inference time, the model scores each candidate concept and ranks all candidates by $s_{CE}(p,c)$.

*Mini-batch construction.* The construction starts by sampling *B* positive pairs ${\left\{(p_{i},c_{i}^{+})\right\}}_{i=1}^{B}$, where B denotes the mini-batch size. For each sampled pair, we sample K negative concepts $\left\{c_{i,1}^{-},\ldots ,c_{i,K}^{-}\right\}$ (with K=4 in our experiments), yielding a per-pair candidate set $C_{\text{cand},i}=\{c_{i}^{+},c_{i,1}^{-},\ldots ,c_{i,K}^{-}\}.$ The negative sampling is performed on each epoch, which allows the model to be exposed to more negative concepts during the training phase. Unlike the bi-encoder, we do not use in-batch negatives for the cross-encoder: optimization only involves the (K+1) pairs formed for each sampled positive pair.

*Training objectives.* We consider two variants:

- *Pointwise.* The pointwise cross-encoder is trained as a binary classifier over passage–concept pairs. For each passage $p_{i}$, we consider its per-passage candidate set $C_{\text{cand},i}=\{c_{i}^{+},c_{i,1}^{-},\ldots ,c_{i,K}^{-}\}$ and compute logits $s_{i,0}=s_{\text{CE}}(p_{i},c_{i}^{+})$ and $s_{i,j}=s_{\text{CE}}(p_{i},c_{i,j}^{-})$ for j=1,…,K. Let σ(·) denote the sigmoid function. We use the binary cross-entropy loss
  (9) $$\ell_{\text{BCE}}(s,y)=-y\;\text{log}\;\sigma (s)-(1-y)\;\text{log}\;\left(1-\sigma (s)\right),$$
  and minimize
  (10) $$L_{\text{CE}}^{\text{pt}}=\sum_{i=1}^{B}[\ell_{\text{BCE}}(s_{\text{CE}}(p_{i},c_{i}^{+}),1)+\sum_{j=1}^{K}\ell_{\text{BCE}}(s_{\text{CE}}(p_{i},c_{i,j}^{-}),0)].$$
- *Listwise.* The listwise cross-encoder directly optimizes the ranking within $C_{\text{cand},i}$. For each $p_{i}$, we compute scores ${\left\{s(p_{i},c)\right\}}_{c\in C_{\text{cand},i}}$ and minimize a softmax cross-entropy loss:
  (11) $$L_{\text{CE}}^{\text{list}}=-\sum_{i=1}^{B}\;\text{log}\;\frac{\;\text{exp}\;(s_{\text{CE}}(p_{i},c_{i}^{+}))}{\sum_{c\in C_{\text{cand},i}}\;\text{exp}\;(s_{\text{CE}}(p_{i},c))}.$$

We evaluate both pointwise (PCER) and listwise (LCER) cross-encoders under one-stage and two-stage inference settings. Based on the results in Section 4.1, LCER is adopted as the default reranker in subsequent ENR experiments.

### 2.3 Inference settings

We evaluate all models under a one-stage setting, where a single model outputs $\hat{Y}(p)$ over the full ontology C (Fig. 2). We further evaluate a two-stage **retrieve-rerank** setting for MA-BCR: a first-stage model produces a passage-specific candidate set $C_{p}\subseteq C$, and a cross-encoder reranker scores only the candidates $c\in C_{p}$.

#### 2.3.1 One-stage inference

In the one-stage setting, a single model produces $\hat{Y}(p)$ with respect to the full ontology C. For ranking-based models (Bi-Encoder and Cross-Encoder), we perform full-ontology scoring, that is, compute s(p,c) for all c∈C and obtain $\hat{Y}(p)$ by applying a decision threshold calibrated to maximize the development-set F1. For XR-Transformer, we form $\hat{Y}(p)$ by thresholding its concept-level scores using the same calibration procedure. For MA-COIR, which generates index sequences rather than concept-level scores, we control concept generation via beam-search decoding: we select a beam width $W^{*}\in \{1,\ldots ,10\}$ on the development set by maximizing the F1 score of the concept set obtained from the top-1 decoded sequence (We found that generating more sequences only brings lower F1 scores). The generated indices in the top-1 sequence are mapped to concepts via an index-concept lookup table, and we take the resulting concept set as $\hat{Y}(p)$.

#### 2.3.2 Two-stage inference

In the two-stage setting, a first-stage model produces a passage-specific candidate set $C_{p}\subseteq C$ intended to cover the true concepts with high recall, and a second-stage cross-encoder reranker scores only candidates $c\in C_{p}$ to output the final prediction set $\hat{Y}(p)\subseteq C_{p}$. To systematically compare candidate-generation strategies for MA-BCR, we pair PCER/LCER with different first-stage recognizers (MA-COIR, XR-Transformer, and Bi-Encoder) under multiple candidate construction schemes, as reported in Table 2.

**Table 2:** Performance of generative, classification-based, and ranking-based models under one-stage and two-stage inference on MM-HPO and MM-GO.

**Inference architecture**			**MM-HPO**				**MM-GO**			
1st-stage	C-Selection	2nd-stage	Pre (%)	Rec (%)	F1 (%)	C-Rec (%)	Pre (%)	Rec (%)	F1 (%)	C-Rec (%)
MA-COIR	Beam-tuned, Top-1	–	43.4	19.8	27.2	–	29.0	12.0	16.9	–
XR-Transformer	Thresholded	–	45.8	30.8	36.9	–	**37.3**	26.6	31.1	–
Bi-Encoder	Thresholded	–	34.7	36.7	35.7	–	19.4	19.3	19.4	–
PCER	Thresholded	–	39.1	44.7	41.7	–	20.2	27.8	23.4	–
LCER	Thresholded	–	**54.2**	**54.5**	**54.4**	–	31.3	**37.2**	**34.0**	–
MA-COIR	Beam-tuned, Top-1	PCER	70.0	19.7	30.8	19.8	48.3	11.2	18.2	12.0
XR-Transformer	Thresholded	PCER	71.1	29.3	41.5	30.8	49.6	25.4	33.6	26.6
Bi-encoder	Thresholded	PCER	61.1	35.7	45.0	36.7	**56.0**	14.7	23.3	19.3
MA-COIR	Beam-10	PCER	68.3	25.0	36.6	26.4	45.0	13.0	20.2	15.7
XR-Transformer	Top-100	PCER	60.3	40.9	48.7	45.0	32.4	33.2	32.8	43.8
Bi-encoder	Top-100	PCER	55.8	56.7	**56.2**	**86.6**	34.3	**45.7**	39.1	**74.6**
MA-COIR	Beam-tuned, Top-1	LCER	**75.0**	19.4	30.8	19.8	53.0	11.6	19.0	12.0
XR-Transformer	Thresholded	LCER	71.5	29.7	41.9	30.8	49.2	25.3	33.4	26.6
Bi-encoder	Thresholded	LCER	62.3	34.4	44.4	36.7	48.8	16.3	24.5	19.3
MA-COIR	Beam-10	LCER	63.6	25.9	36.8	26.4	39.5	14.3	21.0	15.7
XR-Transformer	Top-100	LCER	62.8	42.1	50.4	45.0	39.0	34.2	36.4	43.8
Bi-encoder	Top-100	LCER	51.7	**60.1**	55.6	**86.6**	41.6	43.6	**42.6**	**74.6**

In the one-stage setting, classifiers and rankers score against the full concept set C, and final predictions are obtained according to the concept-selection rule *C-Selection* (e.g. development-set-tuned thresholding for score-based models). In the two-stage setting, the first stage constructs a passage-specific candidate set $C_{p}$ according to *C-Selection*, which is subsequently reranked by either a pointwise cross-encoder ranker (PCER) or a listwise cross-encoder ranker (LCER). *C-Rec* denotes the recall of $C_{p}$. For MA-COIR (*beam-tuned, top-1*), beam width is selected on the development set to maximize Micro-F1 of the top-1 decoded output, and test-time candidates are derived from concepts mapped from the top-1 decoded sequence. Bolded values are the **best** scores in one- or two-stage settings.

*Candidate selection.* For XR-Transformer and Bi-Encoder, we construct $C_{p}$ in either of two ways: (i) *thresholded*, including all concepts whose scores exceed a decision threshold tuned on the development set to maximize Micro-F1; or (ii) *top-L*, taking the top-*L* ranked concepts without thresholding.

For MA-COIR, we consider two variants. In the *beam-tuned (top-1)* variant, we select a beam width $W^{*}\in \{1,\ldots ,10\}$ on the development set by maximizing the F1 score of the top-1 decoded output, and construct $C_{p}$ from the concepts mapped from that top-1 sequence at test time. In the *beam-W* variant, we decode with a fixed beam width *W*, map the top-*W* decoded sequences to concepts, and take the union of the mapped concepts as $C_{p}$.

### 2.4 Error-Aware Negative-Enhanced ranking (ENR)

As illustrated in Fig. 1, ENR enhances LCER training by combining **gold-derived negatives (GNs)** from lexical retrieval with **error-derived negatives (ENs)** collected from model false positives.

*GN-mining.* For each gold passage-concept pair $\left(p,c^{+}\right)$, we retrieve lexically similar but non-gold concepts using BM25 over concept names to form a GN pool of size $\left|N_{\text{GN}}|\in \{5,10,15,20,25,30,35,40,45,50\right\}$. Because BM25 may return fewer non-zero-scoring concepts than the requested size, the effective GN pool size can be smaller and varies across datasets.

*EN-mining (distribution-matched).* We derive ENs from three trained one-stage recognizers: MA-COIR, XR-Transformer, and the Bi-Encoder. To ensure fair comparison across recognizers, we match **the average number of false positives per passage** across models: we use MA-COIR (Beam-10) as a reference and adjust the thresholds of XR-Transformer and Bi-Encoder so that all three recognizers produce the same average number of false positives per passage on the training set. False positives produced under this matched setting are collected as ENs.

*Negative pool variants.* We evaluate (i) GN-only, (ii) EN-only (single-recognizer or multi-recognizer unions), and (iii) GN+EN pools. When combining GNs and ENs, the number of ENs $\left|N_{\text{EN}}\right|$ is fixed, and we increase the negative pool size |N| by adding more gold-derived lexical negatives. The resulting pool serves only as a reservoir of candidate negatives. During training, we dynamically sample K=4 negatives per positive pair from this pool at each epoch, regardless of the pool size (If |N|≤4, all negatives within the pool are selected during the mini-batch construction). All LCER models are trained with the same listwise contrastive objective and evaluated using the fixed Bi-Encoder (top-100)→LCER retrieve-rerank inference pipeline.

## 3 Experiments

### 3.1 Datasets

We construct two MA-BCR evaluation datasets: **MM-HPO** and **MM-GO**, by reprocessing MedMentions-ST21pv (MM-ST21pv), a widely used benchmark for biomedical entity linking (Mohan and Li 2019). MM-ST21pv provides concept annotations for PubMed abstracts using UMLS concept identifiers from the ST21pv subset of UMLS 2017AA, including concepts that can be mapped to the Human Phenotype Ontology (HPO) and the Gene Ontology (GO).

Each PubMed abstract is treated as a passage *p*, and its annotated UMLS concepts are remapped to ontology identifiers in HPO and GO. We retain only phenotypic abnormality concepts for MM-HPO (18 585 concepts) and biological process concepts for MM-GO (26 036 concepts), and keep instances where at least one target concept is present. Although MM-ST21pv was originally annotated through mention-level concept linking, our reformulation removes mention boundaries and concept-mention associations at both training and inference time, requiring models to predict concepts directly from passages. Dataset statistics are reported in Table 1. Examples are provided in Supplemental Materials B.

**Table 1:** Data statistics of MM-HPO and MM-GO.

Dataset	Set	Passage	Concept	Unique concept
MM-HPO	Train	1312	2720	949
	Development	442	906	455
	Test	417	897	440
	All	2171	4523	1238
MM-GO	Train	1032	2526	746
	Development	336	792	335
	Test	350	760	336
	All	1718	4078	1002

### 3.2 Evaluation protocol

For each passage p, a model predicts a concept set $\hat{Y}(p)\subseteq C$. We report micro-averaged Precision (Pre), Recall (Rec), and F1 on the test set against ground truth Y(p)⊆C. For two-stage inference, we additionally report candidate recall (C-Rec) of the first-stage candidate set $C_{p}$, measuring the proportion of gold concepts Y(p) covered by $C_{p}$.

### 3.3 Implementation details

*MA-COIR and XR-Transformer.* We follow the original architectures and training recipes of Liu *et al.* (2025) for MA-COIR and Zhang *et al.* (2021) for XR-Transformer. MA-COIR is instantiated with BART (facebook/bart-large), and XR-Transformer uses BERT (bert-base-uncased) as the text encoder (Devlin *et al.* 2019, Lewis *et al.* 2019) (We also tested XR-Transformer with SapBERT in place of BERT. SapBERT gave slightly better absolute performance, but did not change the relative ranking of the three paradigms or any of the claims in this paper).

For MA-COIR training, we construct two types of supervision. First, each training passage is paired with its associated gold concept indices, forming the standard passage-to-index generation instances. Second, for every concept appearing in the training set, we additionally create concept-only instances that map the concept name directly to its SSI. This auxiliary concept-to-index pairing provides explicit supervision over the index space and stabilizes decoding. Similarly, we train the XR-Transformer with passage-to-label and concept-to-label instances.

*Ranking models.* For both the bi-encoder and the cross-encoder, we initialize the encoder with SapBERT (Liu *et al.* 2021). The bi-encoder is trained with contrastive learning using hard negatives retrieved by BM25 over gold concept names. For each positive pair $\left(p,c^{+}\right)$, we retrieve the top-2 non-gold concepts as hard negatives, and additionally leverage in-batch negatives. We apply FAISS (Douze *et al.* 2024) to accelerate bi-encoder inference. The embeddings z of all concepts within C are pre-calculated and stored, and then we can retrieve high-scoring concepts by computing the embedding of the given passage p and using the nearest-neighbour search function provided by FAISS. The cross-encoder is trained with the mini-batch construction described in Section 2.2.4.

*Inference backbone selection.* We first evaluate multiple one-stage and two-stage inference pipelines by pairing different first-stage recognizers with the cross-encoder ranker, as reported in Table 2. Based on this comparison, we identify the Bi-Encoder (Top-100)→LCER configuration as the strongest inference backbone. This backbone is fixed in all subsequent ENR experiments to ensure that performance differences arise solely from the choice of negative construction strategies for LCER training.

*Negative construction for LCER training.* For inference backbone selection, LCER is trained with 20 gold-derived negatives (GNs) obtained via BM25. Only one-run results are reported. To study error-derived negatives (ENs), we replace the GN pool with alternative negative pools following the EN-mining protocol in Section 2.4. ENs from different recognizers are matched in quantity to control for negative pool size, and are evaluated both in isolation and in combination with GNs. Results are averaged over two runs with different random seeds (Average Micro-F1 differences across all settings between two runs are ≤1.57%.).

*Hyperparameters and model selection.* For the bi-encoder, we use a learning rate of $2\times 10^{-5}$, train for up to 5 epochs, set the mini-batch size to 16, and sample K=2 hard negatives per positive pair (We train the bi-encoder for 5 epochs to match the default XR-Transformer setting, ensuring a controlled comparison across paradigms. Using 20 epochs will be slightly better than using 5.). Model checkpoints are selected on the development set using accuracy@1 over a pool consisting of one positive concept and its top-20 BM25-retrieved negatives.

For the cross-encoder, we use the same learning rate ($2\times 10^{-5}$), train for up to 10 epochs with a mini-batch size of 8, and dynamically sample K=4 negatives per positive pair. We apply early stopping with a patience of 3 epochs. Unless otherwise specified, cross-encoder checkpoints are selected using the same development-set accuracy@1 criterion as the bi-encoder. For the ENR experiments, where LCER is used as a dedicated reranker, checkpoints are instead selected according to development-set nDCG@100, which directly measures ranking quality over candidate lists.

All experiments are conducted on a single NVIDIA A100 GPU.

## 4 Results

### 4.1 Inference settings

Table 2 compares one-stage and two-stage inference across generative, classification-based, and ranking-based paradigms for MA-BCR.

*One-stage inference.* In the one-stage setting, models predict concepts directly over the full ontology. Among all one-stage approaches, LCER achieves the highest accuracy (MM-HPO: F1 = 54.4%; MM-GO: F1 = 34.0%), reflecting the benefit of cross-attention for modelling fine-grained passage–concept interactions. Compared with PCER (MM-HPO: F1 = 41.7%; MM-GO: F1 = 23.4%), its additional gains suggest that listwise optimization is particularly effective when ranking against the full ontology. However, full-ontology scoring with cross-encoders is prohibitively expensive in practice (e.g. ∼250 seconds per passage on MM-GO in our setup), limiting its applicability.

Among the remaining one-stage models, XR-Transformer provides the strongest accuracy–efficiency trade-off, outperforming MA-COIR and Bi-Encoder despite lacking explicit contrastive negative supervision.

*Two-stage inference (candidate generation* → *reranking).* In the two-stage setting, overall effectiveness depends critically on the recall of the first-stage candidate set. Under the same candidate budget (Top-100), Bi-Encoder achieves substantially higher candidate recall than XR-Transformer (C-Rec 86.6% vs. 45.0% on MM-HPO; 74.6% vs. 43.8% on MM-GO). As a result, Bi-Encoder-based pipelines consistently achieve the strongest end-to-end performance.

We additionally compare PCER and LCER as second-stage rerankers across all candidate generators. While LCER consistently outperforms PCER in the one-stage setting, the gap becomes much smaller after candidate restriction. For Bi-Encoder (Top-100), PCER and LCER achieve comparable performance on MM-HPO (56.2% vs. 55.6%), whereas LCER retains an advantage on MM-GO (42.6% vs. 39.1%).

These results highlight a complementary division of labour in retrieve-rerank architectures: contrastively trained bi-encoders are effective at retrieving gold concepts into a compact shortlist, while the cross-encoder provides the discriminative power needed to separate gold concepts from hard confounders. Based on this systematic comparison, we adopt Bi-Encoder (Top-100)→LCER configuration as the inference backbone in all subsequent ENR experiments.

### 4.2 False positive analysis

We manually analyse false positives (FPs) using the passage-grounded diagnostic workflow and taxonomy of Liu *et al.* (2026). Because MM-ST21pv was originally annotated for mention-level concept linking, some gold concepts lack passage-level support, while some passage-supported concepts are absent from the original annotations. To account for these discrepancies, we first construct a *passage-supported reference set* by revising the dataset-provided gold annotations. Specifically, we remove gold concepts that lack passage-level support and add passage-supported but unannotated concepts surfaced by model predictions and manually verified against the passage text (denoted as **Additions**). The resulting reference set serves as the reference annotation for error analysis; predictions excluded by this reference set are treated as true FPs and categorized into five FP types: *granularity mismatch* (E-1), *semantic scope shift* (E-2), *context over-generalization* (E-3), *lexical triggering* (E-4), and *inferential overreach* (E-5).

**Figure 3:**
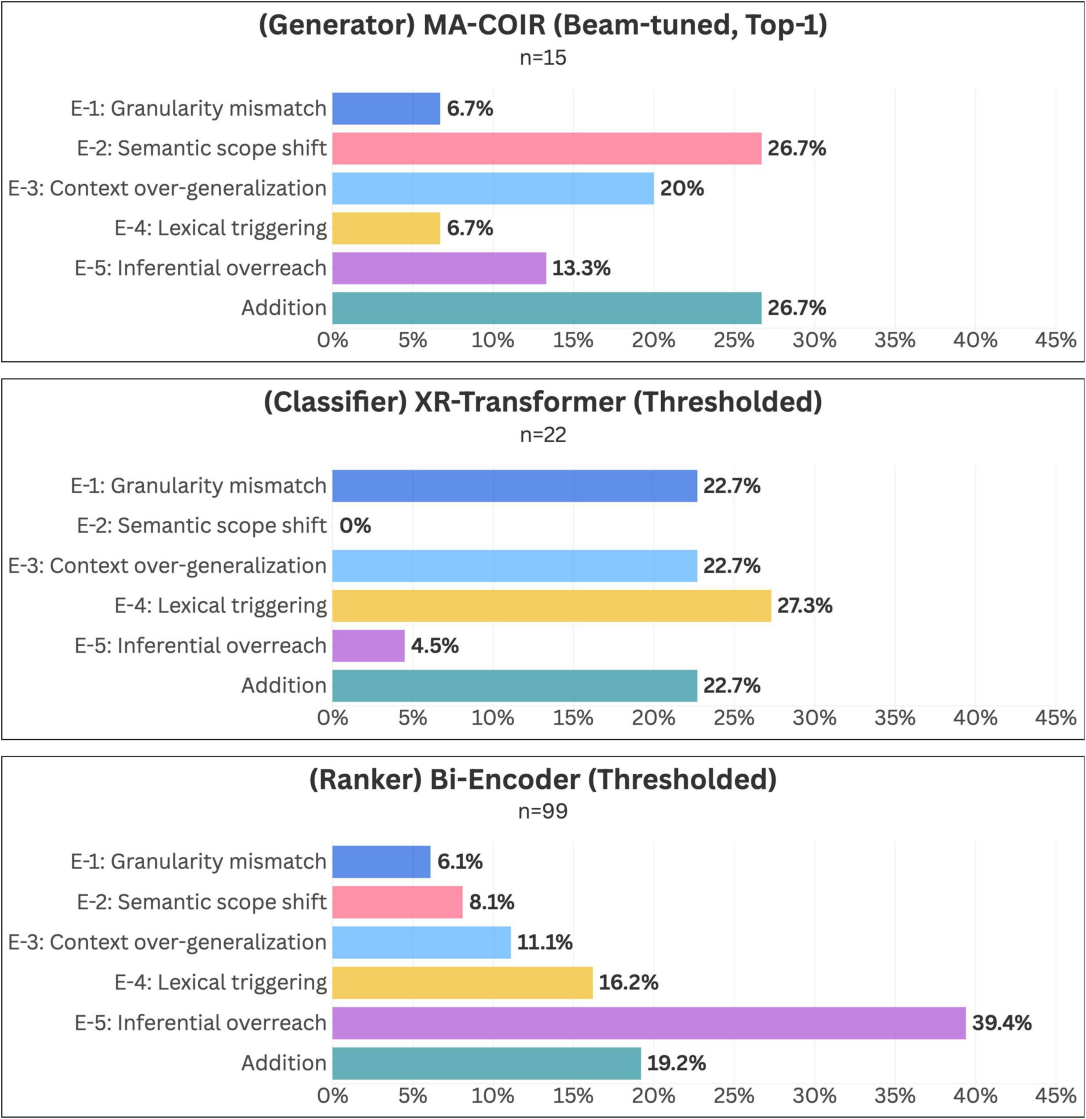
Distribution of initial false positives provided by different models on 20 randomly sampled MM-GO development instances. “Additions” are passage-supported concepts missing from the dataset gold; remaining false positives are grouped into five types.

We conduct a case study on 20 randomly sampled MM-GO development instances (Fig. 3). Examples are provided in Supplemental Materials B. Among 46 dataset gold concepts, 15.2% (7/46) of gold concepts lack passage support. All three models surface many **Additions** (26.7%/22.7%/19.2% for MA-COIR/XR-Transformer/Bi-Encoder), consistent with the mismatch between mention-level annotations and passage-level MA-BCR evaluation.

After reconciling model predictions against the passage-supported reference set, the remaining true FPs exhibit clear, paradigm-specific profiles: MA-COIR produces few FPs (*n* = 15) mainly from semantic scope shift (E-2) and context over-generalization (E-3); XR-Transformer is dominated by lexical triggering (E-4) with non-trivial E-1/E-3; and the Bi-Encoder yields the most FPs (*n* = 99), largely inferential overreach (E-5), reflecting confusions among closely related concepts.

Our analysis shows that false positives from different paradigms exhibit **markedly different error patterns**. This observation motivates ENR: errors produced by multiple one-stage recognizers provide informative, model-realistic confounders for training cross-encoder rerankers. The addition rates above are from a dev-set diagnosis; ENs are mined on the training set under a distribution-matched setting (≈3 FPs per passage) and therefore primarily serve as in-distribution hard negatives under the dataset annotation regime.

### 4.3 Effectiveness of the ENR framework

**Figure 4:**
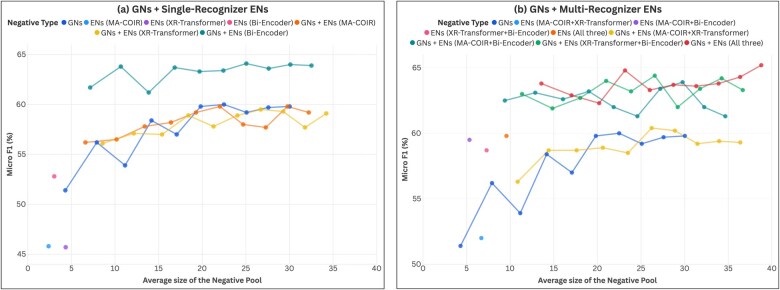
Effect of error-derived negatives (ENs) on LCER performance on MM-HPO under varying negative pool sizes. All settings are evaluated using the same two-stage inference pipeline, where a bi-encoder retriever generates the top-100 candidate concepts and an LCER performs reranking. (a) Results using ENs derived from a single recognizer, evaluated alone or in combination with gold-derived negatives (GNs). (b) Results using ENs aggregated from multiple recognizers, highlighting the effect of error diversity.

**Figure 5:**
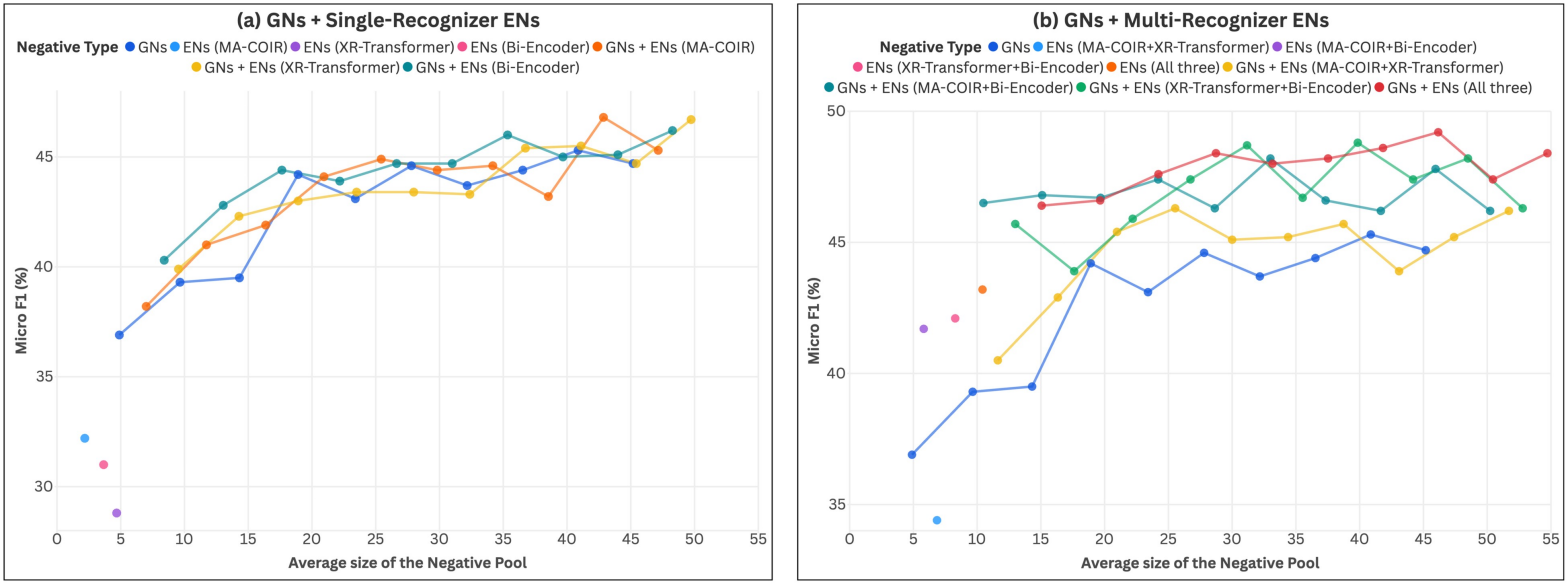
Effect of error-derived negatives (ENs) on LCER performance on MM-GO under varying negative pool sizes. All settings are evaluated using the same two-stage inference pipeline, where a bi-encoder retriever generates the top-100 candidate concepts and an LCER performs reranking. (a) Results using ENs derived from a single recognizer, evaluated alone or in combination with gold-derived negatives (GNs). (b) Results using ENs aggregated from multiple recognizers, highlighting the effect of error diversity.

Figures 4 and 5 illustrate the effect of error-derived negatives (ENs) on ranking performance under varying negative pool sizes. The x-axis denotes the average size of the negative pool per gold passage-concept pair, while the y-axis reports Micro-F1. Specific numerical results can be found in the Supplementary Materials A.

*Single-recognizer ENs.*  Figures 4(a) and 5(a) report results where ENs are derived from a single recognizer and optionally combined with GNs. The number of ENs alone does not directly correlate with final performance. Although the XR-Transformer produces the largest number of ENs per gold passage–concept pair under the distribution-matched setting (4.34 on MM-HPO and 4.68 on MM-GO, compared to 2.38/2.18 for MA-COIR and 3.03/3.66 for Bi-Encoder), its ENs consistently yield the weakest results.

On MM-HPO, Bi-Encoder ENs achieve the strongest performance (52.8%). We attribute this advantage to the characteristics of the HPO scenario, where the number of semantically or lexically similar distractors is relatively limited—when retrieving up to 50 BM25 negatives per gold concept, only 30 candidates on average have non-zero similarity scores, compared to 45 in MM-GO. In this setting, Bi-Encoder errors provide fine-grained semantic confusions that are otherwise difficult to obtain. On MM-GO, where concept semantics are more complex and densely structured, the performance gap between EN sources becomes smaller, suggesting that no single recognizer provides sufficient confounder diversity.

*GNs + Single-recognizer ENs.* Combining ENs with GNs improves performance, but the magnitude of improvement depends on the EN source. On MM-HPO, incorporating Bi-Encoder ENs yields the largest gain, improving F1 from 60.0% (GN-only) to 64.1%. This is consistent with the strong EN-only performance of Bi-Encoder ENs, indicating that Bi-Encoder errors provide informative and complementary signals beyond lexical negatives. In contrast, on MM-GO, all single-recognizer ENs yield only marginal improvements over GN-only training (46.2–46.8% vs. 45.3%), suggesting that a single EN source—even when combined with lexical negatives—is insufficient to substantially enrich the negative pool in domains with higher semantic complexity and denser concept spaces.

*Multi-recognizer ENs.*  Figures 4(b) and 5(b) further examine the effect of incorporating ENs from multiple recognizers. Simply aggregating ENs from different models does not guarantee improved performance: combining MA-COIR and XR-Transformer ENs—without Bi-Encoder ENs—yields poor results on both datasets (52.0% and 34.4%), even falling below the performance obtained using a small GN pool of size 5. This indicates that effective negative pools must also contain negatives that remain sufficiently close to the gold concepts in semantic or lexical space, which are largely absent when Bi-Encoder ENs and GNs are excluded. In contrast, combining ENs from all three recognizers substantially improves performance (59.8% and 43.2%), demonstrating that their error patterns provide complementary supervision signals.

*GNs + Multi-recognizer ENs.* When multi-recognizer ENs are combined with GNs, all configurations containing Bi-Encoder ENs consistently outperform GN-only training. The full ENR configuration integrating ENs from all three recognizers achieves the best performance on both datasets, reaching 65.2% Micro-F1 on MM-HPO and 49.2% on MM-GO, corresponding to an 8.6% relative improvement over the best GN-only setting (60.0% and 45.3%).

Overall, these results show that the gains of ENR arise not simply from increasing the number of negatives. Rather, effective negative pools require both semantically or lexically proximate confounders and diverse error patterns. Under this condition, multi-recognizer ENs complement lexical negatives and substantially improve cross-encoder ranking performance.

## 5 Discussion

*Inference strategies for MA-BCR.* A key contribution of this work is the first systematic comparison of generative, classification-based, and ranking-based paradigms for MA-BCR. Our results show that retrieve-rerank inference provides the most effective balance between scalability and accuracy. While full-ontology cross-encoder ranking achieves the strongest one-stage performance, its computational cost is prohibitive in practice. Conversely, end-to-end performance in two-stage inference is primarily determined by candidate recall, with Bi-Encoder (Top-100)→LCER consistently providing the strongest results. These findings highlight candidate generation as a key bottleneck for future MA-BCR systems.

*Diversity beyond hardness.* Hard negative mining has been extensively studied across retrieval, entity-centric prediction, and large-label classification tasks. Representative examples include lexical-retrieval negatives (BM25), dense-retrieval negatives (DPR; Karpukhin *et al.* 2020), retrieval-augmented systems such as REALM (Guu *et al.* 2020), self-mined dense negatives (ANCE; Xiong *et al.* 2020), teacher-filtered negatives (RocketQA; Qu *et al.* 2021), and retrieved entity candidates used in entity linking systems BLINK (Wu *et al.* 2020) and GENRE (De Cao *et al.* 2020). Although these approaches differ in how hard negatives are constructed, their primary goal is to expose models to difficult confounders that lie close to the decision boundary.

Our results suggest that hardness alone may not be sufficient. ENR aggregates false positives produced by heterogeneous recognizers spanning generative, classification-based, and ranking-based paradigms. False-positive analysis shows that these paradigms exhibit qualitatively distinct error profiles, ranging from lexical triggering to inferential overreach, indicating that the resulting confounders capture complementary forms of model confusion. Because these confounders originate from model errors, they remain sufficiently close to the decision boundary. The resulting performance gains support the use of heterogeneous model confusions as an effective source of supervision for large-label biomedical concept recognition. More broadly, these findings suggest that good hard negatives should not only be difficult but also diverse enough to cover complementary regions of the decision boundary.

*Implications for ontology-driven bioinformatics.* ENR establishes a new performance level for MA-BCR on both evaluated benchmarks. Beyond these gains, our results suggest that progress in biomedical concept recognition is not solely driven by more expressive architectures, but also by how effectively limited supervision is transformed into informative training signals. In particular, diverse yet sufficiently proximate confounders appear to provide more effective training signals than difficult negatives alone. This observation may be especially relevant for ontology-driven bioinformatics tasks, where large candidate spaces and limited annotations make effective supervision a persistent challenge.

## 6 Conclusion

We presented the first systematic comparison of generative, classification-based, and ranking-based paradigms for mention-agnostic biomedical concept recognition (MA-BCR). Our results show that a bi-encoder→listwise cross-encoder retrieve-rerank architecture provides a robust and scalable inference backbone for ontology-scale MA-BCR. Building on these findings, we proposed ENR, an error-aware negative-enhanced ranking framework that leverages false positives from heterogeneous recognizers as training signals without increasing inference cost. ENR achieves substantial performance gains on human phenotype abnormality and biological process concept recognition, demonstrating the value of diverse and informative negatives for large-label biomedical concept recognition with limited supervision.

## Supplementary Material

btag495_Supplementary_Data
