# Supplementary material for: Biomedical concept recognition with error-aware negative-enhanced ranking framework
Source: Bioinformatics. 2026 Jul 3;42(7):btag495. doi: 10.1093/bioinformatics/btag495 (PMC13401478; doi:10.1093/bioinformatics/btag495)
Supplement: btag495_Supplementary_Data [file btag495_supplementary_data.pdf]

Supplemental Materials

A. Results achieved by different negative strategies.

Table 1 and Table 2 list numeric results achieved with all negative strategies evaluated using the same two-stage inference pipeline, where a bi-encoder retriever generates the top 100 candidate concepts and a listwise cross-encoder ranker (LCER) performs re-ranking. These results are specific numbers displayed in Figures 4 and 5 of the main paper.

Table 1: Results on MM-HPO.

GN refers to gold-derived negative, while EN refers to error-derived negative, provided fine-tuned first-stage concept recognizers. F1 scores are calculated in a micro average setting. Run-1 uses a random seed of 66, while Run-2 uses 42.

* Average size of the Negative Pool = Average number of Negatives per Gold Pair.

Negative Type	Average size of the Negative Pool *	Average number of Negatives per Passage	Run-1 F1 (%)	Run-2 F1 (%)	Average F1 (%)
GNs	4.3	8.7	52.2	50.5	51.4
GNs	7.9	15.8	56.2	56.2	56.2
GNs	11.2	22.2	53.5	54.3	53.9
GNs	14.2	28.1	58.2	58.5	58.4
GNs	17.1	33.8	58.0	56.0	57.0
GNs	19.8	39.1	60.8	58.7	59.8
GNs	22.5	44.2	59.9	60.1	60.0
GNs	25.1	49.2	60.6	57.8	59.2
GNs	27.6	54.1	56.7	62.7	59.7
GNs	30.0	58.8	59.6	60.1	59.8
ENs (MA-COIR)	2.4	3.2	45.3	46.3	45.8
ENs (XR-Transformer)	4.3	3.2	46.6	44.9	45.7
ENs (Bi-Encoder)	3.0	3.2	53.9	51.6	52.8
GNs + ENs (MA-COIR)	6.6	11.7	55.4	57.0	56.2
GNs + ENs (MA-COIR)	10.2	18.7	56.7	56.4	56.5
GNs + ENs (MA-COIR)	13.4	25.1	58.5	57.0	57.8
GNs + ENs (MA-COIR)	16.4	31.0	59.3	57.0	58.2
GNs + ENs (MA-COIR)	19.3	36.6	59.7	58.7	59.2
GNs + ENs (MA-COIR)	22.0	41.9	59.1	60.5	59.8
GNs + ENs (MA-COIR)	24.7	47.0	59.9	56.0	58.0
GNs + ENs (MA-COIR)	27.3	52.0	57.5	57.9	57.7
GNs + ENs (MA-COIR)	29.8	56.8	63.0	56.6	59.8
GNs + ENs (MA-COIR)	32.2	61.5	56.8	61.6	59.2
GNs + ENs (XR-Transformer)	8.6	11.8	55.4	56.9	56.1
GNs + ENs (XR-Transformer)	12.2	18.9	59.1	55.2	57.1
GNs + ENs (XR-Transformer)	15.4	25.3	57.2	56.8	57.0
GNs + ENs (XR-Transformer)	18.4	31.2	60.5	57.3	58.9
GNs + ENs (XR-Transformer)	21.3	36.8	58.3	57.3	57.8
GNs + ENs (XR-Transformer)	24.0	42.1	60.0	57.7	58.9
GNs + ENs (XR-Transformer)	26.7	47.3	60.2	58.9	59.5
GNs + ENs (XR-Transformer)	29.3	52.3	59.2	59.5	59.3
GNs + ENs (XR-Transformer)	31.8	57.1	57.4	58.1	57.7
GNs + ENs (XR-Transformer)	34.2	61.8	60.2	58.0	59.1
GNs + ENs (Bi-Encoder)	7.2	11.7	60.6	62.7	61.7

**GNs + ENs (Bi-Encoder)**	10.7	18.8	64.1	63.5	63.8
**GNs + ENs (Bi-Encoder)**	13.9	25.2	58.4	64.1	61.2
**GNs + ENs (Bi-Encoder)**	16.8	31.0	63.8	63.6	63.7
**GNs + ENs (Bi-Encoder)**	19.7	36.6	61.7	64.9	63.3
**GNs + ENs (Bi-Encoder)**	22.4	41.9	63.8	62.9	63.4
**GNs + ENs (Bi-Encoder)**	25.1	47.1	63.8	64.4	64.1
**GNs + ENs (Bi-Encoder)**	27.6	52.1	63.8	63.4	63.6
**GNs + ENs (Bi-Encoder)**	30.1	56.9	63.9	64.1	64.0
**GNs + ENs (Bi-Encoder)**	32.5	61.6	64.6	63.2	63.9
**ENs (MA-COIR+XR-Transformer)**	6.7	6.3	52.6	51.3	52.0
**ENs (MA-COIR+Bi-Encoder)**	5.3	6.3	61.1	57.8	59.5
**ENs (XR-Transformer+Bi-Encoder)**	7.3	6.3	59.2	58.2	58.7
**ENs (All three)**	9.6	9.4	59.7	59.9	59.8
**GNs + ENs (MA-COIR+XR-Transformer)**	10.8	14.7	56.4	56.2	56.3
**GNs + ENs (MA-COIR+XR-Transformer)**	14.4	21.8	59.8	57.6	58.7
**GNs + ENs (MA-COIR+XR-Transformer)**	17.6	28.1	58.5	58.9	58.7
**GNs + ENs (MA-COIR+XR-Transformer)**	20.6	34.0	59.6	58.1	58.9
**GNs + ENs (MA-COIR+XR-Transformer)**	23.5	39.6	59.7	57.3	58.5
**GNs + ENs (MA-COIR+XR-Transformer)**	26.2	44.9	59.4	61.4	60.4
**GNs + ENs (MA-COIR+XR-Transformer)**	28.9	50.0	59.5	60.9	60.2
**GNs + ENs (MA-COIR+XR-Transformer)**	31.5	55.0	59.5	58.9	59.2
**GNs + ENs (MA-COIR+XR-Transformer)**	33.9	59.8	59.6	59.1	59.4
**GNs + ENs (MA-COIR+XR-Transformer)**	36.4	64.5	60.2	58.4	59.3
**GNs + ENs (MA-COIR+Bi-Encoder)**	9.4	14.7	63.4	61.6	62.5
**GNs + ENs (MA-COIR+Bi-Encoder)**	12.9	21.7	62.2	64.0	63.1
**GNs + ENs (MA-COIR+Bi-Encoder)**	16.0	28.0	62.0	63.2	62.6
**GNs + ENs (MA-COIR+Bi-Encoder)**	19.0	33.9	64.5	61.8	63.2
**GNs + ENs (MA-COIR+Bi-Encoder)**	21.9	39.4	62.6	61.5	62.0
**GNs + ENs (MA-COIR+Bi-Encoder)**	24.6	44.7	61.9	60.8	61.3
**GNs + ENs (MA-COIR+Bi-Encoder)**	27.2	49.8	64.5	62.3	63.4
**GNs + ENs (MA-COIR+Bi-Encoder)**	29.8	54.8	63.9	64.0	63.9
**GNs + ENs (MA-COIR+Bi-Encoder)**	32.2	59.6	63.6	60.4	62.0
**GNs + ENs (MA-COIR+Bi-Encoder)**	34.7	64.3	61.8	60.8	61.3
**GNs + ENs (XR-Transformer+Bi-Encoder)**	11.4	14.8	62.4	63.5	63.0
**GNs + ENs (XR-Transformer+Bi-Encoder)**	14.8	21.9	62.6	61.3	61.9
**GNs + ENs (XR-Transformer+Bi-Encoder)**	18.0	28.2	62.1	63.4	62.7
**GNs + ENs (XR-Transformer+Bi-Encoder)**	21.0	34.1	63.0	65.0	64.0
**GNs + ENs (XR-Transformer+Bi-Encoder)**	23.9	39.7	64.3	62.2	63.2
**GNs + ENs (XR-Transformer+Bi-Encoder)**	26.6	45.0	65.4	63.5	64.4
**GNs + ENs (XR-Transformer+Bi-Encoder)**	29.2	50.1	63.0	61.0	62.0
**GNs + ENs (XR-Transformer+Bi-Encoder)**	31.8	55.1	64.3	62.6	63.4
**GNs + ENs (XR-Transformer+Bi-Encoder)**	34.2	59.9	64.5	63.8	64.2
**GNs + ENs (XR-Transformer+Bi-Encoder)**	36.7	64.6	65.4	61.2	63.3
**GNs + ENs (All three)**	13.6	17.7	64.3	63.3	63.8
**GNs + ENs (All three)**	17.0	24.7	63.6	62.2	62.9
**GNs + ENs (All three)**	20.2	31.0	61.5	63.0	62.3
**GNs + ENs (All three)**	23.2	36.9	63.9	65.7	64.8
**GNs + ENs (All three)**	26.0	42.5	62.4	64.3	63.3
**GNs + ENs (All three)**	28.7	47.7	63.6	63.8	63.7
**GNs + ENs (All three)**	31.3	52.8	63.9	63.4	63.6
**GNs + ENs (All three)**	33.9	57.8	62.9	64.7	63.8
**GNs + ENs (All three)**	36.4	62.6	64.6	64.0	64.3

GNs + ENs (All three)	38.8	67.3	65.3	65.2	65.2

Table 2: Results on MM-GO.

GN refers to gold-derived negative, while EN refers to error-derived negative, provided fine-tuned first-stage concept recognizers. F1 scores are calculated in a micro average setting. Run-1 uses a random seed of 66, while Run-2 uses 42.

* Average size of the Negative Pool = Average number of Negatives per Gold Pair.

Negative Type	Average size of the Negative Pool *	Average number of Negatives per Passage	Run-1 F1 (%)	Run-2 F1 (%)	Average F1 (%)
GNs	4.9	11.7	36.3	37.5	36.9
GNs	9.7	22.9	40.5	38.0	39.3
GNs	14.3	33.9	39.0	40.1	39.5
GNs	18.9	44.7	44.0	44.4	44.2
GNs	23.4	55.0	42.7	43.5	43.1
GNs	27.8	65.2	44.2	45.1	44.6
GNs	32.2	75.3	44.0	43.4	43.7
GNs	36.5	85.3	44.8	44.1	44.4
GNs	40.9	95.2	44.0	46.7	45.3
GNs	45.2	105.0	44.4	45.1	44.7
ENs (MA-COIR)	2.2	3.2	30.8	33.5	32.2
ENs (XR-Transformer)	4.7	3.2	30.4	27.2	28.8
ENs (Bi-Encoder)	3.7	3.2	29.2	32.8	31.0
GNs + ENs (MA-COIR)	7.0	14.7	39.2	37.2	38.2
GNs + ENs (MA-COIR)	11.7	25.8	41.3	40.7	41.0
GNs + ENs (MA-COIR)	16.4	36.8	41.6	42.2	41.9
GNs + ENs (MA-COIR)	20.9	47.5	43.1	45.0	44.1
GNs + ENs (MA-COIR)	25.4	57.8	46.4	43.3	44.9
GNs + ENs (MA-COIR)	29.8	68.0	43.5	45.3	44.4
GNs + ENs (MA-COIR)	34.2	78.0	45.7	43.4	44.6
GNs + ENs (MA-COIR)	38.5	88.1	45.3	41.1	43.2
GNs + ENs (MA-COIR)	42.9	97.9	45.8	47.7	46.8
GNs + ENs (MA-COIR)	47.2	107.7	43.3	47.2	45.3
GNs + ENs (XR-Transformer)	9.5	14.8	39.4	40.4	39.9
GNs + ENs (XR-Transformer)	14.3	26.0	42.1	42.5	42.3
GNs + ENs (XR-Transformer)	18.9	36.9	41.0	45.1	43.0
GNs + ENs (XR-Transformer)	23.5	47.7	43.8	43.0	43.4
GNs + ENs (XR-Transformer)	28.0	58.1	43.3	43.4	43.4
GNs + ENs (XR-Transformer)	32.4	68.2	42.6	44.0	43.3
GNs + ENs (XR-Transformer)	36.7	78.3	46.1	44.7	45.4
GNs + ENs (XR-Transformer)	41.1	88.3	46.9	44.1	45.5
GNs + ENs (XR-Transformer)	45.4	98.2	44.1	45.4	44.7
GNs + ENs (XR-Transformer)	49.7	108.0	45.8	47.6	46.7
GNs + ENs (Bi-Encoder)	8.4	17.7	40.5	40.1	40.3
GNs + ENs (Bi-Encoder)	13.0	28.8	42.4	43.2	42.8
GNs + ENs (Bi-Encoder)	17.6	39.7	45.7	43.2	44.4
GNs + ENs (Bi-Encoder)	22.2	50.3	43.1	44.7	43.9
GNs + ENs (Bi-Encoder)	26.6	60.7	44.6	44.9	44.7
GNs + ENs (Bi-Encoder)	31.0	70.8	44.8	44.5	44.7
GNs + ENs (Bi-Encoder)	35.3	80.8	46.6	45.3	46.0
GNs + ENs (Bi-Encoder)	39.7	90.9	43.6	46.4	45.0

**GNs + ENs (Bi-Encoder)**	44.0	100.7	45.8	44.3	45.1
**GNs + ENs (Bi-Encoder)**	48.3	110.5	47.1	45.3	46.2
**ENs (MA-COIR+XR-Transformer)**	6.8	6.3	33.2	35.6	34.4
**ENs (MA-COIR+Bi-Encoder)**	5.8	6.3	42.1	41.3	41.7
**ENs (XR-Transformer+Bi-Encoder)**	8.3	6.3	42.8	41.3	42.1
**ENs (All three)**	10.4	9.4	41.9	44.5	43.2
**GNs + ENs (MA-COIR+XR-Transformer)**	11.6	17.7	40.1	40.9	40.5
**GNs + ENs (MA-COIR+XR-Transformer)**	16.3	28.9	42.0	43.8	42.9
**GNs + ENs (MA-COIR+XR-Transformer)**	21.0	39.8	46.0	44.9	45.4
**GNs + ENs (MA-COIR+XR-Transformer)**	25.5	50.5	46.6	46.0	46.3
**GNs + ENs (MA-COIR+XR-Transformer)**	30.0	60.8	43.5	46.7	45.1
**GNs + ENs (MA-COIR+XR-Transformer)**	34.4	71.0	44.9	45.4	45.2
**GNs + ENs (MA-COIR+XR-Transformer)**	38.7	81.0	44.8	46.5	45.7
**GNs + ENs (MA-COIR+XR-Transformer)**	43.1	91.0	44.4	43.3	43.9
**GNs + ENs (MA-COIR+XR-Transformer)**	47.4	100.9	46.3	44.2	45.2
**GNs + ENs (MA-COIR+XR-Transformer)**	51.7	110.7	46.3	46.1	46.2
**GNs + ENs (MA-COIR+Bi-Encoder)**	10.5	14.7	45.9	47.2	46.5
**GNs + ENs (MA-COIR+Bi-Encoder)**	15.1	25.9	48.5	45.2	46.8
**GNs + ENs (MA-COIR+Bi-Encoder)**	19.7	36.8	46.9	46.5	46.7
**GNs + ENs (MA-COIR+Bi-Encoder)**	24.2	47.5	47.7	47.1	47.4
**GNs + ENs (MA-COIR+Bi-Encoder)**	28.6	57.9	46.5	46.0	46.3
**GNs + ENs (MA-COIR+Bi-Encoder)**	33.0	68.1	48.3	48.0	48.2
**GNs + ENs (MA-COIR+Bi-Encoder)**	37.3	78.1	46.0	47.2	46.6
**GNs + ENs (MA-COIR+Bi-Encoder)**	41.7	88.2	46.9	45.5	46.2
**GNs + ENs (MA-COIR+Bi-Encoder)**	46.0	98.1	47.4	48.1	47.8
**GNs + ENs (MA-COIR+Bi-Encoder)**	50.2	107.8	47.3	45.1	46.2
**GNs + ENs (XR-Transformer+Bi-Encoder)**	13.0	17.8	44.8	46.6	45.7
**GNs + ENs (XR-Transformer+Bi-Encoder)**	17.6	28.9	44.2	43.6	43.9
**GNs + ENs (XR-Transformer+Bi-Encoder)**	22.2	39.9	45.6	46.1	45.9
**GNs + ENs (XR-Transformer+Bi-Encoder)**	26.7	50.5	46.3	48.4	47.4
**GNs + ENs (XR-Transformer+Bi-Encoder)**	31.2	60.9	48.3	49.0	48.7
**GNs + ENs (XR-Transformer+Bi-Encoder)**	35.5	71.1	45.5	47.8	46.7
**GNs + ENs (XR-Transformer+Bi-Encoder)**	39.9	81.1	48.6	49.0	48.8
**GNs + ENs (XR-Transformer+Bi-Encoder)**	44.2	91.1	47.9	46.9	47.4
**GNs + ENs (XR-Transformer+Bi-Encoder)**	48.5	101.0	47.9	48.5	48.2
**GNs + ENs (XR-Transformer+Bi-Encoder)**	52.8	110.8	46.5	46.1	46.3
**GNs + ENs (All three)**	15.1	20.7	45.9	46.9	46.4
**GNs + ENs (All three)**	19.7	31.8	46.5	46.7	46.6
**GNs + ENs (All three)**	24.2	42.7	48.0	47.3	47.6
**GNs + ENs (All three)**	28.7	53.3	49.5	47.4	48.4
**GNs + ENs (All three)**	33.2	63.6	47.6	48.4	48.0
**GNs + ENs (All three)**	37.5	73.8	47.6	48.7	48.2
**GNs + ENs (All three)**	41.8	83.8	49.1	48.1	48.6
**GNs + ENs (All three)**	46.2	93.8	49.0	49.4	49.2
**GNs + ENs (All three)**	50.4	103.7	47.0	47.9	47.4
**GNs + ENs (All three)**	54.7	113.4	49.3	47.5	48.4

B. Data examples of MM-HPO and MM-GO.

We provide 3 instances from MM-HPO in Table 3.

As described in Section 3.2 of the main paper, a false-positive analysis is conducted on 20 development instances from MM-GO. Thus, we provide 3 instances from MM-GO, with the false-positive analyses in Table 4.

Table 3: Example instances of MM-HPO.

**Item**	**Detail**
	**Instance 1 - 25763772**
Passage	DCTN4 as a modifier of chronic Pseudomonas aeruginosa infection in cystic fibrosis.<br>Pseudomonas aeruginosa (Pa) infection in cystic fibrosis (CF) patients is associated with worse long-term **pulmonary disease** and shorter survival, and chronic Pa infection (CPA) is associated with reduced lung function, faster rate of lung decline, increased rates of exacerbations and shorter survival. By using exome sequencing and extreme phenotype design, it was recently shown that isoforms of dynactin 4 (DCTN4) may influence Pa infection in CF, leading to worse respiratory disease. The purpose of this study was to investigate the role of DCTN4 missense variants on Pa infection incidence, age at first Pa infection and chronic Pa infection incidence in a cohort of adult CF patients from a single centre. Polymerase chain reaction and direct sequencing were used to screen DNA samples for DCTN4 variants. A total of 121 adult CF patients from the Cochin Hospital CF centre have been included, all of them carrying two CFTR defects: 103 developed at least 1 **pulmonary infection** with Pa, and 68 patients of them had CPA. DCTN4 variants were identified in 24% (29/121) CF patients with Pa infection and in only 17% (3/18) CF patients with no Pa infection. Of the patients with CPA, 29% (20/68) had DCTN4 missense variants vs 23% (8/35) in patients without CPA. Interestingly, p.Tyr263Cys tend to be more frequently observed in CF patients with CPA than in patients without CPA (4/68 vs 0/35), and DCTN4 missense variants tend to be more frequent in male CF patients with CPA bearing two class II mutations than in male CF patients without CPA bearing two class II mutations (P = 0.06). Our observations reinforce that DCTN4 missense variants, especially p.Tyr263Cys, may be involved in the pathogenesis of CPA in male CF.
Gold concept – UMLS (ID: Mention)	(C0024115: pulmonary disease), (C0876973, pulmonary infection)
Gold concept – HPO (ID: Concept)	(HP_0002088: Abnormal lung morphology), (HP_0006532: Recurrent pneumonia)
	**Instance 2 - 26406200**
Passage	Seated maximum flexion: An alternative to standing maximum flexion for determining presence of flexion - relaxation?<br>The flexion - relaxation phenomenon (FRP) in standing is a specific and sensitive diagnostic tool for **low back pain**. Seated flexion as an alternative could be beneficial for certain populations, yet the behavior of the trunk extensors during seated maximum flexion compared to standing flexion remains unclear. Compare FRP occurrences and spine angles between seated and standing flexion postures in three levels of the erector spinae muscles. Thirty-one participants free of **back pain** performed seated and standing maximum trunk flexion. Electromyographical signals were recorded from the bilateral lumbar (L3), lower-thoracic (T9), and upper-thoracic (T4) erector spinae and assessed for the occurrence of FRP. Spine angles corresponding to FRP onset and cessation were determined, and FRP occurrences and angles were compared between posture and muscle. FRP occurrence was similar in standing and seated maximum flexion across all muscles, with the lumbar muscles showing the greatest consistency. Standing FRP onset and cessation angles were consistently greater than the corresponding seated FRP angles. Considering the similar number of FRP occurrences, seated maximum flexion may constitute an objective criterion for **low back pain** diagnosis. Future work should seek to confirm the utility of this test in individuals with **low back pain**.
Gold concept – UMLS (ID: Mention)	(C0024031: low back pain), (C0004604: back pain)
Gold concept – HPO (ID: Concept)	(HP_0003419: Low back pain), (HP_0003418: Back pain)
	**Instance 3 - 26867927**
Passage	Radiofrequency ablation of posteroseptal accessory pathways associated with **coronary sinus diverticula**.<br>Posteroseptal accessory pathways may be associated with a **coronary sinus (CS) diverticulum**. Our purpose was to describe the clinical characteristics, mapping and ablation of these pathways. This was a retrospective study of all patients who underwent ablation of posteroseptal accessory pathways in a single centre. Patients with a **diverticulum of the CS** or one of its tributaries were included in group I, while the other patients formed group II. Clinical presentation, ablation procedure and outcome were compared between the two groups. A total of 51 patients were included, 16 in group I and 35 in group II. There were no significant differences in age or sex distribution. **Atrial fibrillation** (**AF**) and previous unsuccessful ablation were more common in group I. A negative delta wave in lead II was the ECG finding with best sensitivity and specificity for the presence of a diverticulum. A pathway potential was common at the successful site in group I, and the interval between local ventricular electrogram and delta wave onset was shorter (19.5 ± 8 vs 33.1 ± 7.6 ms, $p < 0.001$). There was a trend toward lower procedural success rate and higher recurrence rate in group I, although this was not significant. **CS diverticula** should be suspected in patients with manifest posteroseptal accessory pathways who have a previous failed ablation, documented **AF** or typical electrocardiographic signs. A discrete potential is frequently seen at the successful site, but the local ventricular electrogram is not as early as in other accessory pathways.
Gold concept – UMLS (ID: Mention)	(C3163894: coronary sinus diverticula), (C3163894: coronary sinus (CS) diverticulum), (C3163894: diverticulum of the CS), (C0004238: Atrial fibrillation), (C0004238: AF), (C3163894: CS diverticula)
Gold concept – HPO (ID: Concept)	(HP_0011644: Coronary sinus diverticulum), (HP_0005110: Atrial fibrillation)

Table 4: Example instances of MM-GO.

Initial false positives are manually analysed with the process we described in the Section 3.2. Gold concepts without textual support, true false positives and passage-supported but unannotated concepts (Additions) are provided.

**Item**	**Detail**
**Instance 1 – 28179308**	
Passage	Renieramycin M Attenuates Cancer Stem Cell -like Phenotypes in H460 Lung Cancer Cells. Cancer stem cells (CSCs) are a subpopulation of cancer cells that possess **self-renewal** and **differentiation** capacities. CSCs contribute to drug-resistance, cancer recurrence and metastasis, thus development of CSC -targeted therapeutic strategies has recently received significant attention in cancer research. In this study, the potential efficacy of renieramycin M (RM) isolated from the sponge Xestospongia species, was examined against lung CSCs. Colony and spheroid formation assays, as well as western blotting analysis of lung CSC protein markers were employed to determine the CSC -like phenotypes of H460 lung cancer cells after treatment with RM at non-toxic concentrations. RM treatment reduced significantly colony and spheroid formation of H460 cells. Moreover, the CSC markers CD133, CD44 and ALDH1A1 of CSC -enriched H460 cells were reduced significantly following RM treatment. RM could be a potent anti-metastatic agent by suppressing lung CSC -like phenotypes in H460 cells.
Gold concept – UMLS (ID: Mention)	(C1155711: self-renewal), (C0007589: differentiation)
Gold concept – GO (ID: Concept)	(GO_0017145: stem cell division), (GO_0030154: cell differentiation)
Unsupported gold (ID: Concept)	(GO_0017145: stem cell division), (GO_0030154: cell differentiation)
False positive – MA-COIR (ID: Concept, Error Type/Addition)	None
False positive – XR-Transformer (ID: Concept, Error Type/Addition)	(GO_0010467: gene expression, Lexical triggering), (GO_0008283: cell population proliferation, Inferential overreach), (GO_0050789: regulation of biological process, Context over-generalization)
False positive – Bi-Encoder (ID: Concept, Error Type/Addition)	(GO_0048863: stem cell differentiation, Lexical triggering), (GO_0048864: stem cell development, Context over-generalization), (GO_0048867: stem cell fate determination, Inferential overreach), (GO_0048866: stem cell fate specification, Inferential overreach), (GO_0048865: stem cell fate commitment, Inferential overreach), (GO_0019827: stem cell population maintenance, Inferential overreach), (GO_0072089: stem cell proliferation, Inferential overreach), (GO_0043696: dedifferentiation, Inferential overreach), (GO_0043697: cell dedifferentiation, Inferential overreach), (GO_2000738: positive regulation of stem cell differentiation, Inferential overreach), (GO_0060290: transdifferentiation, Inferential overreach), (GO_0001709: cell fate determination, Inferential overreach), (GO_0045165: cell fate commitment, Inferential overreach)
**Instance 2 – 27744219**	
Passage	Biomimetic whitlockite inorganic nanoparticles -mediated in situ **remodeling** and rapid **bone regeneration**. **Bone remodeling** process relies on complex signaling pathway between osteoblasts and osteoclasts and control mechanisms to achieve **homeostasis** of their **growth** and **differentiation**. Despite previous achievements in understanding complicated signaling pathways between cells and bone extracellular matrices during **bone remodeling** process, a role of local ionic concentration remains to be elucidated. Here, we demonstrate that synthetic whitlockite (WH: Ca18Mg2(HPO4)2(PO4)12) nanoparticles can recapitulate early-stage of **bone regeneration** through stimulating **osteogenic differentiation**, prohibiting osteoclastic activity, and transforming into mechanically enhanced hydroxyapatite (HAP)- neo bone tissues by continuous supply of PO4(3-) and Mg(2+) under physiological conditions. In addition, based on their structural analysis, the dynamic phase transformation from WH into HAP contributed as a key factor for rapid **bone regeneration** with denser hierarchical neo-bone structure. Our findings suggest a groundbreaking concept of ' living bone minerals ' that actively **communicate** with the surrounding system to induce self-healing, while previous notions about bone minerals have been limited to passive products of cellular mineralization.
Gold concept – UMLS (ID: Mention)	(C0085268: remodeling), (C0005972: bone regeneration), (C0085268: Bone remodeling), (C0019868: homeostasis), (C0007595: growth), (C0007589: differentiation), (C0085268: bone remodeling), (C0007589: osteogenic differentiation), (C0007582: communicate)
Gold concept – GO (ID: Concept)	(GO_0046849: bone remodeling), (GO_1990523: bone regeneration), (GO_0042592: homeostatic process), (GO_0016049: cell growth), (GO_0030154: cell differentiation), (GO_0007154: cell communication)
Unsupported gold (ID: Concept)	None
False positive – MA-COIR (ID: Concept, Error Type/Addition)	(GO_0007165: signal transduction, Context over-generalization), (GO_0045779: negative regulation of bone resorption, **Addition**)
False positive – XR-Transformer (ID: Concept, Error Type/Addition)	(GO_0001503: ossification, Context over-generalization), (GO_0030282: bone mineralization, Context over-generalization)
False positive – Bi-Encoder (ID: Concept, Error Type/Addition)	(GO_0001503: ossification, Context over-generalization), (GO_0030282: bone mineralization, Context over-generalization), (GO_0001649: osteoblast differentiation, **Addition**), (GO_0060348: bone development, Semantic scope shift), (GO_0098868: bone growth, Context over-generalization), (GO_0002076: osteoblast development, Granularity mismatch), (GO_0060349: bone morphogenesis, Inferential overreach)

Instance – 27829875	
Passage	Mildly raised tricuspid regurgitant velocity 2.5-3.0 m/s in pregnant women with sickle cell disease is not associated with poor obstetric outcome - An observational cross-sectional study.<br>Pulmonary hypertension is associated with 36% mortality in pregnancy, and 6-10% of patients with sickle cell disease have pulmonary hypertension. Tricuspid regurgitant velocity ⩾2.5 m/s on echocardiography is a well validated means of screening for pulmonary hypertension in the non-pregnant population. This is a pilot study to determine if this is a useful non-invasive screening test for pulmonary hypertension in **pregnancy**, and whether raised tricuspid regurgitant velocity ⩾2.5 m/s was associated with poor outcomes. This is a cross-sectional study over a five-year period in a tertiary referral centre with a specialised multidisciplinary clinic for pregnant women with sickle cell disease. Women with sickle cell disease, no prior pulmonary hypertension and singleton **pregnancies** who had echocardiography with a measurable tricuspid regurgitant velocity in **pregnancy** were included. There were 34 **pregnancies**, of which eight had tricuspid regurgitant velocity ⩾2.5 m/s. There were no significant differences in their characteristics, sickle cell -related complications or medical co-morbidities. The women with tricuspid regurgitant velocity ⩾2.5 m/s had similar obstetric and perinatal outcomes as those with a tricuspid regurgitant velocity <2.5 m/s.
Gold concept – UMLS<br>(ID: Mention)	(C0032961: pregnancy), (C0032961: pregnancies)
Gold concept – GO<br>(ID: Concept)	(GO_0007565: female pregnancy)
Unsupported gold<br>(ID: Concept)	None
False positive – MA-COIR<br>(ID: Concept, Error Type/Addition)	None
False positive – XR-Transformer<br>(ID: Concept, Error Type/Addition)	None
False positive – Bi-Encoder<br>(ID: Concept, Error Type/Addition)	(GO_0007567: parturition, Lexical triggering)
